# Maturation of protective immunity induced by SIVΔnef correlates with differential expression of transcription factors in SIV-specific CD8+ T cells

**DOI:** 10.1186/1742-4690-9-S2-P33

**Published:** 2012-09-13

**Authors:** JM Billingsley, PA Rajakumar, NC Salisch, YV Kuzmichev, HS Hong, MA Connole, RK Reeves, H Kang, W Li, RP Johnson

**Affiliations:** 1 New England Primate Research Center Harvard Medical School, Southborough, MA, USA; 2 University of Massachusetts Medical School, Worcester, MA, USA

## Background

Protective immunity against vaginal challenge in SIVΔnef-vaccinated macaques develops at 20 weeks after vaccination, whereas the magnitude of SIV-specific CD8+ T cell responses peaks at 5 weeks. SIV-specific CD8+ T cells phenotypically mature from week 5 to 20, as characterized by upregulation of CCR7 and CD127, suggesting that the quality of the CD8+ T cell response may correlate with protection.

## Methods

Highly parallel qRT-PCR was used to characterize the expression of 21 transcription factors (TFs) in T cells sorted into naïve, central, transitional, and effector memory subsets, and in SIV Gag CM9 and Tat SL8-specific CD8+ T cells obtained at wk5 and wk20 after SIV239Δnef vaccination.

## Results

Unsupervised clustering organized T cell samples into groups concordant with cell surface phenotype. SIV-specific CD8+ cells segregated into wk5 and wk20 clusters. 11 of 21 TFs were expressed at significantly different levels at wk20 than at wk5. Wk20 cells exhibited increased levels of TFs associated with both quiescence and maintenance of effector function. Furthermore, 7 TFs were significantly differentially expressed between SIV Gag and SIV Tat-specific wk20 populations. Principal component analysis suggests the Gag-specific cells may be more effector-like and the Tat-specific cells more transitional or central memory-like.

## Conclusion

Our data indicate distinct transcriptional profiles of different memory T cell subsets and clear differences between wk5 and wk20 SIV-specific CD8+ T cell transcriptomes. The mature wk 20 CD8+ T cell response temporally correlated with protection is characterized by the expression of transcription factors associated with both central memory and effector memory T cells. Additionally, wk20 Gag-specific cells exhibit a more effector-like expression profile than Tat-specific cells, which is consistent with the Tat epitope exhibiting more rapid CTL escape kinetics than the Gag epitope. Analysis of transcription factor expression therefore provides a valuable complement to the analysis of memory cell differentiation based on classical phenotypic markers.

